# A Complex Network Theory-Based Modeling Framework for Unmanned Aerial Vehicle Swarms

**DOI:** 10.3390/s18103434

**Published:** 2018-10-12

**Authors:** Lizhi Wang, Dawei Lu, Yuan Zhang, Xiaohong Wang

**Affiliations:** 1 Unmanned System Institute, Beihang University, Beijing 100191, China; wanglizhi@buaa.edu.cn; 2 Key Laboratory of Advanced Technology of Intelligent Unmanned Flight System of Ministry of Industry and Information Technology, Beijing 100191, China; 3 School of Reliability and Systems Engineering, Beihang University, Beijing 100191, China; buaaldw@buaa.edu.cn (D.L.); zyuan@buaa.edu.cn (Y.Z.)

**Keywords:** unmanned aerial vehicle (UAV) swarms, system modeling, complex network theory

## Abstract

Unmanned aerial vehicle (UAV) swarms is an emerging technology that will significantly expand the application areas and open up new possibilities for UAVs, while also presenting new requirements for the robustness and reliability of the UAV swarming system. However, its complex and dynamic characteristics make it extremely challenging and uncertain to model such a system. In this study, to reach a full understanding of the swarming system, a modeling framework based on complex network theory is presented. First, the scope of work is identified from the point of view of control algorithms considering the dynamics and research novelty of the development of UAV swarming control strategy and three control structures consisting of three interdependent network layers are proposed. Second, three algorithms that systematically build the modeling framework considering all characteristics of the system are also developed. Finally, some network measurements are introduced by adjusting the fundamental ones into the UAV swarming system. The proposed framework is applied to a case study to illustrate the visualization models and estimate the statistical characteristics of the proposed networks with static and dynamic topology analysis. Furthermore, a simple demonstration of the robustness evaluation of the network is also presented. The networks obtained from this framework can be used to further analyze the robustness or reliability of a UAV swarming system in a high-confrontation battlefield environment the effect of cascading failure in ad-hoc network on system.

## 1. Introduction

Unmanned aerial vehicles (UAV) swarms have gradually attracted increasing attention because of their higher flexibility, efficiency, and reliability compared to the use of a single UAV [1]. They have extensive application potential in various areas, such as surveillance, coordinated rescue missions, and, most importantly, in combat missions [2]. These capabilities have presented new requirements of UAV swarming systems. Especially, for such a system, any damage in it or the loss of control will pose risks to the mission or public safety. In recent years, the existing literature has studied a number of problems in this field. One promising study is the remote control of such swarm-based systems and many useful communication-related methods are proposed to support their deployment [3,4,5,6,7]. For example, Basso presented a network infrastructure by extending the existing Micro Air Vehicle Link protocol to support the coordinated operation of UAV swarms and its control by a single ground control station [8]. Beyond that, the robustness and reliability of such system is also an important issue. For a new control algorithm, robustness is often highlighted as the key distinguishing feature of UAV swarms compared with systems that rely on traditional approaches to multi-UAV coordination [9]. In many cases, a system must be dependable and display a high degree of tolerance to faults before it can be deployed in real-world scenarios [9]. In particular, in the future informatization battlefield, UAV swarms must accomplish a variety of cooperative tasks in complex and changeable environments and guarantee a high task-completion rate under high-confrontation scenarios. However, designing an efficient model to estimate the robustness and reliability of a UAV swarming system is quite challenging, because the complexity of the overall system increases quickly and dramatically as the number of vehicles in the system increases; furthermore, the system structure may have to dynamically adjust or reconnect when under attack [10]. Moreover, the interdependence between physical and communication networks makes it easier to trigger a cascading failure and reduce the reliability of the system. Therefore, to conduct a further analysis of such a system, it is extremely important to build a model that can describe all of its characteristics.

In the scientific literature on the analysis and modeling of complex systems such as UAV swarming systems, in the reliability field there are some key methods, including agent-based models, the Petri-net method, system dynamics, complex-network theory, and other modeling methods. Agent-based modeling is the concept of using multi-agent systems to search for explanatory insight into the collective behavior of agents obeying simple rules, typically in natural systems [11]. This method abstracts the system into a number of autonomous entities and establishes a system model by analyzing the interaction between multiple agents [12]. It is engineering-oriented and has applications in various fields. For example, Bompard used the agent-based method to model the cyber inter-dependencies between information infrastructure and a power system and assess the relevant information impacts [13]. Kaegi adopted the agent-based method for reliability analysis to analyze maintenance strategies of a complex emergent system [14]. The Petri-net method is one of several mathematical modeling languages for the description of distributed systems and the most popular method for discrete event dynamic systems [15]. Using the Petri-net-based approach, Wang provided a Petri-net representation of the dynamic behaviors and hierarchical structure of a train control system [15]. Chen modeled a reconfigurable soft-core processor system with a Petri-net [16]. System dynamics approaches use feedback loops, stocks, and flows to model the dynamic behavior of complex systems. They were first introduced by Forrester in 1961 to analyze the complexity of dynamic systems [17]. Applying this method, Mirchi studied the effect of interactions among the subsystems on the overall behavior of a system and applied it to a water resources system [18]. Walters analyzed the interaction among multi-dimensional components and drivers in production sustainability with a system dynamics model [19]. Complex network theory has proven to be a fertile ground for the modeling of complex systems. One of the main appeals of this approach lies in its power to identify universal properties in the structure of connections among the elementary units of a system [20]. Based on complex network theory, a system can be described by a network in which nodes represent the components and link the physical or logical connections among them [21]. This method has been widely applied in power grids [22], infrastructures [23], transportation systems [24], manufacturing [25], and other fields. In addition to the above approaches, there are also many other methods used to model complex systems. Tien, for example, developed an algorithm for the Bayesian network modeling of a complex infrastructure system to assess its reliability [26]. Wang presented a modeling framework to analyze the cascade of failure interdependencies between electricity and information infrastructure systems [27].

All of the methods discussed above are quite effective in modeling complex systems, but they may serve different purposes and be suited for specific systems. Among them, the Petri-net method is more applicable to a discrete-event dynamic system and well suited for modeling the concurrent behavior of distributed systems [16]. One weakness of this method is that, if modeling a system in detail or a large-size system, the computation complexity is high [28]. System dynamics originates from system theory, so it can apply to any system. However, the utilization of this method is overwhelmingly dependent on the knowledge of a subject-matter expert and it is a semi-quantitative method [28,29]. As for an agent-based model and complex network theory, although both are quite useful, a UAV swarming system can naturally be mapped into complex networks to describe its characteristics. Moreover, the established network can provide a framework for further estimation of the robustness or reliability of the system and make it quite easy to perform topological analysis.

To this end, in this study complex network theory is adopted to explore the features of a UAV swarm. In doing so, the aim is to achieve a full understanding of the swarming system and provide a modeling framework to build a multi-layer UAV swarming network. This network can be used to further analyze the performance of a UAV swarming system in a high-confrontation battlefield environment and the effect of cascading failure in an ad hoc network on the system.

The rest of this paper is organized as follows: in Section 2, a generalized description of a UAV swarming system is provided and the scope of work identified. In Section 3, the modeling framework, including network representation, modeling algorithm, and network measurements, is presented. In Section 4, a case study is provided to illustrate the visualization models and to estimate the statistical characteristics of the proposed networks. Some conclusions are drawn in Section 5.

## 2. UAV Swarming System

A UAV swarming system is a large-scale complex system with many dynamic and complex characteristics. A comprehensive analysis of such system could be hardly possible without a good understanding of how the system is organized. Therefore, herein a generalized description of a UAV swarming system is provided in Section 2.1. Moreover, for such a system, the scope can vary from small to large scale. To develop a dynamic modeling methodology on such system, it is important to identify the scope of work. In Section 2.2, this question is considered from the point of view of control algorithms, and three typical formations will be discussed.

### 2.1. System Description

A UAV swarming system is, literally, a flock of UAVs that carry different payloads for multiple missions. The system composition could contain numerous components, but, more generally, the vehicle, the payload, and the datalink are the critical ones [30]:

*(a)*  *Vehicle*: The swarming formation consists of vehicles (individual UAVs). Based on the report “Sustaining America’s Precision Strike Advantage” issued by the U.S. Center for Strategic and Budgetary Assessment (CSBA) in 2015, small UAVs will be the main formation vehicle used to consume enemy weapons [31]. Thus, current research has focused on small and low-cost UAVs, such as the DARPA “elf” UAV, the “Coyote” UAV of the LOCUST project, and the “Partridge” UAVs of the U.S. Navy [32,33]. These vehicles are better for swarming owing to their small size, low cost, and test repeatability, among other attributes.
*(b)*  *Payload*: Payload refers to equipment and sensors related to UAV missions. Sensors, radars, camera equipment, and weapons are the most common payloads [34]. In a typical UAV swarming system, the individual UAV limits the variety of payloads it can carry for technical reasons, especially for small UAVs; thus, the payloads are generally integrated with the aircraft [33,35]. As a result, a UAV swarming system may contain UAVs with different payloads when performing missions. For example, for cooperative detection, the system may be equipped with heterogeneous sensors.
*(c)*  *Datalink*: The communication datalink is the basis for the realization of UAV swarm control and the successful implementation of missions. Two kinds of datalinks are commonly used: a traditional datalink and a UAV ad hoc network [36]. The traditional datalink can be further divided into “ground station to UAV” and “ground station to satellite to UAV” links [37]. A communication ad hoc network is the network formed by multiple UAVs. It will be the main communication mode in the future or the next generation of communication datalinks. At present, three kinds of communication ad hoc networks (mobile ad hoc networks, wireless sensor networks, and wireless mesh networks) can be utilized in a UAV swarm owing to their mobility and network topology dynamics, multiple-hop transmission, and self-organization [37,38,39]. Moreover, these networks are rather robust, so that a single-node failure in the network has no effect on the performance of the entire network.

### 2.2. Scope Identification

A UAV swarming system has characteristics such as self-organization, dynamic topology, and random movement. It may constitute a much more effective system than a single vehicle, and the formation control is a critical step in attempting to realize cooperation among UAVs. Thus, to abbreviate the scope of this study and focus on investigating the dynamic behaviors of a UAV swarm, this question is considered from the point of view of a control algorithm since it is the core of the swarming formation of UAVs. Over the last few years, many formation-control algorithms have been put forward in various research communities. They can be classified into two typical types, i.e., behavior-based methods [40,41] and leader-follower-based strategy [40,42]. A behavior-based method is based on the design and description of the behavior of an individual vehicle. The core idea originates from bionics and some famous ones like pigeon flock- and ant colony-based UAV control algorithms have been well studied [43]. The leader-follower-based approach designates some individual vehicles as leaders informed with global trajectory information with the uninformed ones as followers [42]. The followers must sense the leaders’ motion information and adjust their own position and velocity. These two approaches are the most popular ones in state-of-the-art control algorithms. However, the autonomy level increases with technology, the control of a UAV will gradually change from simple remote control to interactive control with human-machine intelligent integration, and eventually develop into a fully autonomous control mode [43]. Then, the future UAV swarming system can truly organize like biological flocking.

**Figure 1:**
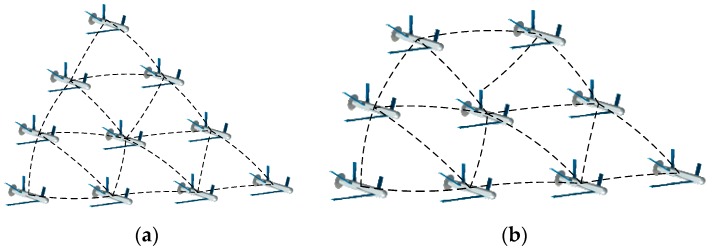
Schematics of typical Structure 1.

Thus, considering the dynamics and research novelty of the development of UAV swarming control algorithms and to ensure this research covers most of the applications both in current and future scenarios, in this paper three structures of a UAV swarming system are chosen based on three types of control algorithm, namely a control structure based on the behavior-based method (Structure 1), on leader-follower strategy (Structure 2), and on autonomous control (Structure 3):

*Structure 1*: Behavior-based methods are inspired by the interactions found in large flocks of birds or honeybees; those based on pigeons flocking have gained extensive attention due to the hierarchical leadership network existing in multiple-pigeon flocks [44]. In the hierarchy, pigeons follow the individuals in the upper ranks and lead the flights of individuals in lower ranks. The organized flight of such flocks in the sky is seemingly a well-rehearsed group dance [45]. Two schematics of such structure are shown in Figure 1.

**Figure 2:**
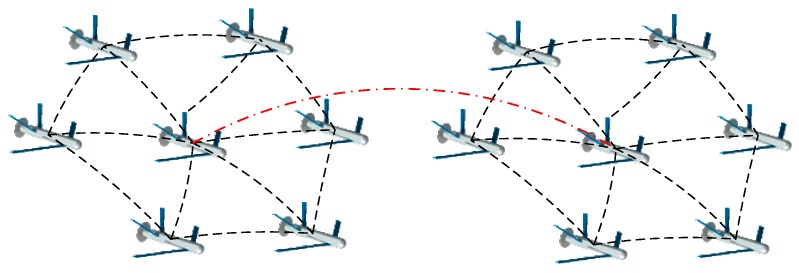
Schematic of typical Structure 2.

*Structure 2*: The control structure based on leader-follower strategy, Structure 2, differs from Structure 1. The leader needs to be designated and the followers need to be defined to follow the leader. If there are more than two leaders, each leader and its followers will form a community. The leaders can communicate with each other while the followers of each community can only communicate with the leaders and the UAVs that are adjacent to them [42]. Moreover, in order to maintain the formation, a temporary leader will be chosen when the leader is attacked. A schematic of a control structure with two leaders is shown in Figure 2.

*Structure 3*: When the autonomy level of the UAV is high enough, an autonomous control structure can be realized by simulating the behavior of the biological swarming [43]. Unlike Structure 1, the multiple UAVs can automatically organize, communicate, and interact with each other as well as coordinate to conduct the mission. The formation of this structure can dynamically adjust based on the mission. 

## 3. Modeling Framework

Here, modeling framework is proposed. Specifically, a complex network scheme is introduced to represent relationships existing in the UAV swarming system in Section 3.1. A modeling algorithm is then proposed in Section 3.2 to build the modeling framework considering all of the characteristics of the system. In Section 3.3, the measurement of the characteristics of the UAV swarming system is presented based on the basic characteristics of complex networks, node degree and degree distribution, clustering coefficient, and average path length, among other properties. 

### 3.1. Network Representation Based on Complex Network

A complex network is a network with some or all topological features, such as self-organization, self-similarity, attractor, small world, and scale-free [46]. Currently, complex networks are being studied in many fields of science [47]. It can be seen the majority of systems in reality can be undoubtedly described by models of complex networks based on the description in Section 1. Therefore, herein a complex network is utilized to describe a UAV swarming system.

To study the complex interaction and dynamic relationship in a UAV swarming system, the system is considered as consisting of three interdependent network layers, i.e., the communication layer, the structure layer, and the mission layer, denoted *G_a_*, *G_b_*, and *G_c_*, respectively. Then, to represent the network topology, three networks are abstracted into graphs based on graph theory.

Suppose a UAV swarming system consisting of *n* vehicles and *m* types of payloads. For each payload, there are *n_i_* (*i* = 1, 2, …, *m*) aircraft, where *n*_1_ + *n*_2_ + …+ *n_m_* = *n.* Then, the three network layers and their interlayer relationships can be described as follows:

(a) Communication Layer

Since the communication in the swarming system is based on an ad hoc network and each aircraft is the communication point, the communication points or vehicles in the network are denoted as nodes and the edges as communication links between points. Then, *G_a_* = (*V_ai_*, *E_aj_*, *W_aj_*), where *V_ai_* is the set of nodes, *V_ai_* = {*V_a_*_1_, *V_a_*_2_ …, *V_an_*}; and *E_aj_* is the set of edges, *E_aj_* = {*E_a_*_1_, *E_a_*_2_, …, *E_aN_*_1_} (*j* = 1, 2, 3, …, *N*_1_*, N*_1_ > *n*). Moreover, the communication network keeps changing, which will affect the communication quality. Thus, the communication layer is defined as a weighted network, with the weight denoted *W_aj_*, where *W_aj_* = {*W_a_*_1_, *W_a_*_2_ …, *W_aN_*_1_}.

(b) Structure layer

The structure layer consists of aircraft, and these aircrafts are denoted as nodes and the edges as the distance between aircraft. As in the communication layer, *G_b_* can be defined as *G_b_* = (*V_bi_*, *E_bj_*, *W_bj_*), where *V_bi_* is the set of nodes, *V_bi_* = {*V_b_*_1_, *V_b_*_2_, …, *V_bn_*}; and *E_bj_* is the set of edges, *E_bj_* = {*E_b_*_1_, *E_b_*_2_, …, *E_bN_*_2_} (*j* = 1, 2, 3, …, *N*_2_*, N*_2_ > *n*). The weight is denoted *W_bj_* = {*W_b_*_1_, *W_b_*_2_*,* …, *W_bN_*_2_}.

(c) Mission layer

The mission layer is comprised of payloads. Generally, individual UAVs can carry different types of payloads. the payloads are denoted as nodes and the edges as the connections between payloads which have the same weights, denoted as *W_c_*. Thus, *G_c_* can be written as *G_c_* = (*V_ci_*, *E_ci_*, *W_c_*), where *V_ci_* is the set of nodes, *V_ci_* = {*V_b_*_1_, *V_b_*_2_, …, *V_cn_*}; and *E_cj_* is the set of edges, *E_cj_* = {*E_c_*_1_, *E_c_*_2_, …, *E_cN_*_3_} (*j* = 1, 2, 3, …, *N*_3_*, N*_3_ > *n*).

(d) Interlayer relationship

**Figure 3:**
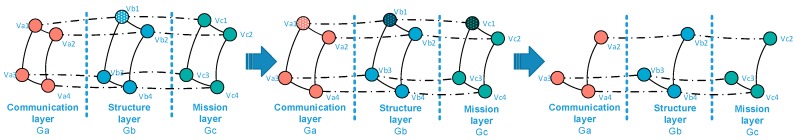
Interlayer relationships between the communication layer and structure layer.

**Figure 4:**
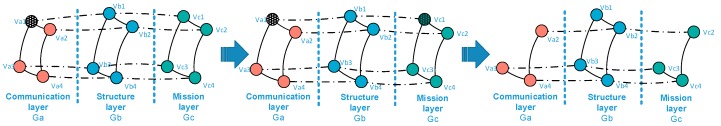
Interlayer relationships between the structure layer and mission layer.

The interlayer relationship in a UAV swarming system is relatively simple, specifically the physical relationship between the nodes of three network layers. *L*(*a − b*)*_i_* (*i* = 1, 2, 3, …, *n*) and *L*(*b − c*)*_i_* are defined as the interlayer relationships between the communication layer and structure layer and between the structure layer and mission layer, respectively. The dynamic relationships are depicted in Figure 3 and Figure 4 which is the removal of nodes in one layer will lead to the removal of nodes in other layers and also the removal of interlayer edges.

**Figure 5:**
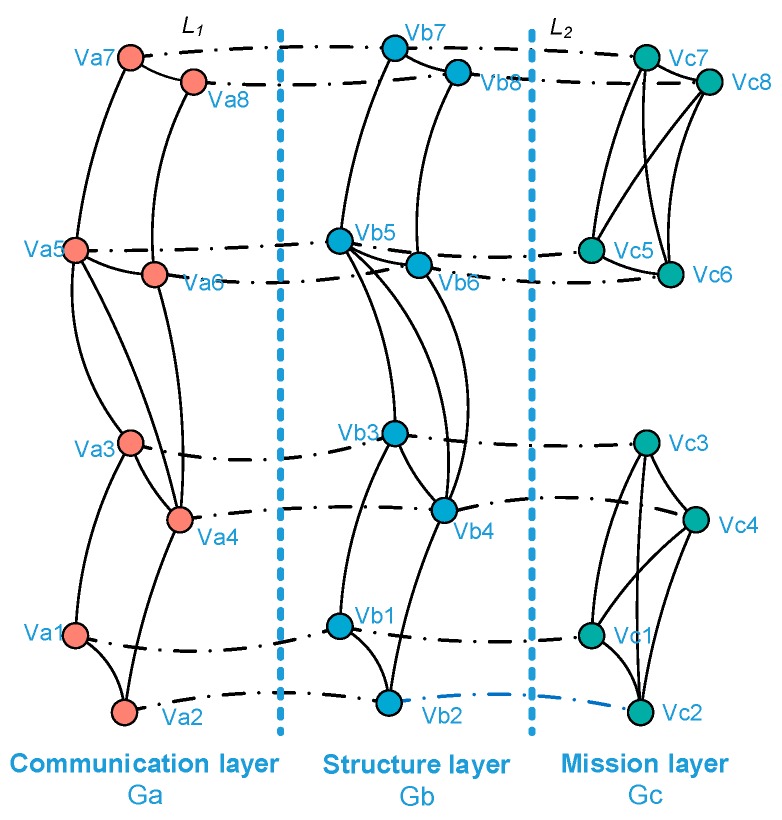
Schematic of network representation.

Based on the description above, a schematic of the network representation based on a complex network is illustrated in Figure 5.

### 3.2. Modeling Algorithm

Based on Section 3.1, three algorithms are developed that systematically build the modeling framework considering all of the characteristics of the system. The algorithms are modular and scalable, since the UAV swarming system can change or reconstruct as necessary based on the mission or modeling objectives. Moreover, the algorithm can be a guideline for modeling a UAV swarming network with programming or other computer tools.

**a. Modeling Algorithm for Structure 1**

Considering the characteristic of the control structure of behavior-based methods, the network topology needs to be defined. Then, the network can be built via the following steps (the pseudocode is shown in Table 1):

1. *Initialization*: Generate *n* nodes and define the number of payload types, *m*; the number of nodes under each payload, *n_i_* (*i* = 1,2, …, *m*); and the number of hierarchy levels, *p*.
2. *Connection:*
  (a) Adds edges between nodes and adjacent nodes in the communication layer and structural layer according to topology;
  (b) Adds edges between every two nodes among *n_i_* nodes;
  (c) Adds edges one-to-one between the communication layer and structure layer and between the structure layer and the mission layer separately;
  (d) Multiple edges and self-loops should not exist.
3. *Weight*: Randomly assign a weight to the edges in the communication layer and structure layer based on mission requirements. The weight of edges in the mission layer should assign the same value.
4. *Output:* After all of the nodes, edges, and weights are generated, output the network.

**b. Modeling Algorithm for Structure 2**

For Structure 2, the core item is the leader and the follower, and one can choose the leader nodes and define the number of followers of each leader. The leader nodes can be denoted *V_j_*. In this paper, the number of leaders is set equal to the number of payload types to simplify the modeling difficulty. Then, one can build the network via the following steps (the pseudocode is shown in Table 2):

1. *Initialization*: Generate *n* nodes and define the number of payload types, *m*, and the number of nodes under each payload, *n_i_* (*i* = 1,2, …, *m*).
2. *Connection:*
  (a) Adds edges between leader nodes and follower nodes in the communication layer and structural layer;
  (b) Adds edges between every follower node and its adjacent nodes;
  (c) Adds edges between every two nodes among *n_i_* nodes;
  (d) Adds edges one-to-one between the communication layer and structure layer and between the structure layer and the mission layer separately;
  (e) Multiple edges and self-loops should not exist.
3. *Weight*: Randomly assign a weight to the edges in the communication layer and structure layer based on mission requirements. The weight of edges in the mission layer should be assigned the same value.
4. *Output*: After all the nodes, edges, and weights are generated, output the network.

**c. Modeling Algorithm for Structure 3**

The formation of Structure 3 can dynamically adjust based on the mission. Considering the dynamic characteristic, edges are randomly added for the communication layer and structure layer instead of using a defined structure. Then, the network can be built via the following steps (the pseudocode is shown in Table 3):

1. *Initialization*: Generate *n* nodes and define the number of payload types, *m*, and the number of nodes under each payload, *n_i_* (*i* = 1, 2, …, *m*).
2. *Connection:*
  (a) Adds edges among nodes and random [*k* − 1,*k* + 1] nodes in the communication layer and the structural layer;
  (b) Adds edges between every two nodes among *n_i_* nodes;
  (c) Adds edges one-to-one between the communication layer and the structure layer and between the structure layer and the mission layer separately;
  (d) Multiple edges and self-loop should not exist.
3. *Weight*: Randomly assign a weight to the edges in the communication layer and the structure layer based on mission requirements. The weight of edges in the mission layer should be assigned the same value.
4. *Output*: After all of the nodes, edges, and weights are generated, output the network.

### 3.3. Network Measurements

Most network studies rely on measures that can characterize relevant topological features to identify the unifying principles and statistical properties commonly found in empirical networks. Therefore, some fundamental measurements need to be discussed to make a characterization of its complex statistical properties, including node degree and degree distribution, clustering coefficient, and average path length, among other properties [48]. In this paper, these measurements are adjusted to a UAV swarming system and presented measurements for a multiple-layer network.

(1) Degree and degree distribution of nodes

The degree of a node in a network is the number of connections it has to other nodes and the degree distribution is the probability distribution of these degrees over the entire network [48].

The node degree in a UAV swarming network can be divided into two parts: (a) the degree of node *V_qi_* calculated from its network layer, which can be denoted *k_qi_*(*G_q_*) (*q* = *a*,*b*,*c*), and (b) the degree calculated by the connections with nodes in another layer, which can be denoted $k_{qi}^{l}$, and the following conditions must be satisfied:

$$\{\begin{array}{c} k_{qi}^{l}=1,V_{qi}\in G_{a}|G_{c}\\ k_{qi}^{l}=2,V_{qi}\in G_{b} \end{array},i=1,2,...N,$$

where *N* is the number of nodes in the UAV swarming network.

Then, the node degree of any node *V_qi_* in the UAV swarming network can be expressed as:

(1) $$k_{qi}=k_{qi}\left(G_{q}\right)+k_{qi}^{l},q=a,b,c;i=1,2,...N.\;$$

The average degree can be written as:

(2) $$k=\frac{1}{3*N}\sum_{q=a}^{c}\sum_{i=1}^{N}k_{qi}.\;$$

The cumulative distribution of degree *P*(*k*) can be defined as:

(3) $$P_{c}\left(k\right)=\sum_{k^{'}\geq k}P(k^{\prime }).\;$$

(2) Clustering coefficient

A clustering coefficient is a measure of the degree to which nodes in a graph tend to cluster together [48]. It can be calculated by the ratio of the possible edges *E_qi_* among nodes *V_qi_* and its *k_qi_* adjacent nodes and the total possible edges *k_qi_* (*k_qi_* − 1)⁄2, i.e.:

(4) $$C_{qi}=\frac{2E_{qi}}{k_{qi}(k_{qi}-1)},\;$$

where $E_{qi}=E_{qi}\left(G_{q}\right)+E_{qi}^{l},q=a,b,c;i=1,2,...N$, and *E_qi_*(*G_q_*) is denoted the connections in the same layer while $E_{qi}^{l}$ is the connection with nodes in different layers, and the following conditions must be satisfied:

$$\{\begin{array}{c} E_{qi}^{l}=1,V_{qi}\in G_{a}|G_{c}\\ E_{qi}^{l}=2,V_{qi}\in G_{b} \end{array},i=1,2,...N.\;$$

Then, the clustering coefficient of the UAV swarming network can be written as:

(5) $$C=\frac{1}{3*N}\sum_{q=a}^{c}\sum_{i=1}^{N}C_{qi}.\;$$

(3) Average path length

The average path length, representing the tightness of connections between nodes in a network, is the average distance between all nodes [48]. It can be defined by the arithmetic mean of the shortest path between any node pairs, i.e.:

(6) $$L=\frac{2}{N(N+1)}\sum_{i\neq j}d_{ij}.\;$$

## 4. Case Study Analysis and Discussion

Here, to illustrate the network modeling process of a UAV swarming system, a case study with the characteristics described in Section 2 is presented. Python NetworkX is utilized to program the modeling algorithm to present the network structure and further analysis of the statistical feature of the network. Note that the network is built through the properties of the UAV swarming system and three types of control structure, so it can be applied in many mission scenarios. However, the validity of a modeling network using complex-network theory is unknown. Therefore, in Section 4.2, the statistical characteristics of the proposed network are compared with real-world networks to test its validity.

### 4.1. Case Study

Suppose a UAV swarming system consisting of 55 small UAVs in which every eleven UAVs carry a certain type of payload to execute a certain mission. Based on the description in Section 2, three scenarios corresponding to the three structures are considered.

**Figure 6:**
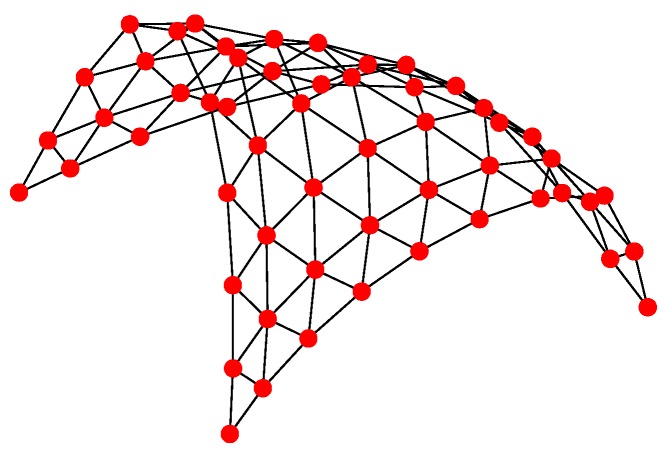
Communication and structure layers of Structure 1.

**Figure 7:**
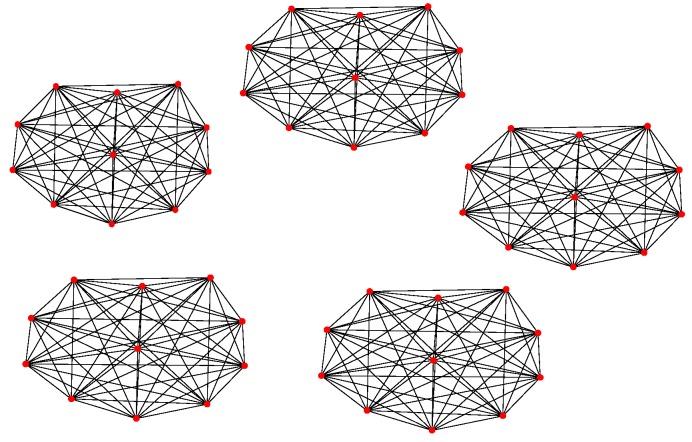
Mission layer of Structure 1.

**Figure 8:**
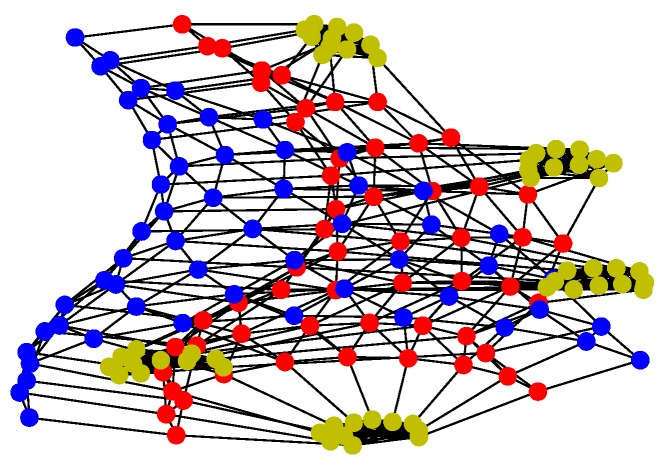
Network of Structure 1.

For Structure 1, the topology of the UAV swarming system is set to be a classic pigeon flock with one “leader.” Then, based on Algorithm 1, the communication layer and the structure layer can be built as shown in Figure 6. The mission layer is shown in Figure 7. Finally, the network for the control structure of behavior-based methods is depicted in Figure 8. 

**Figure 9:**
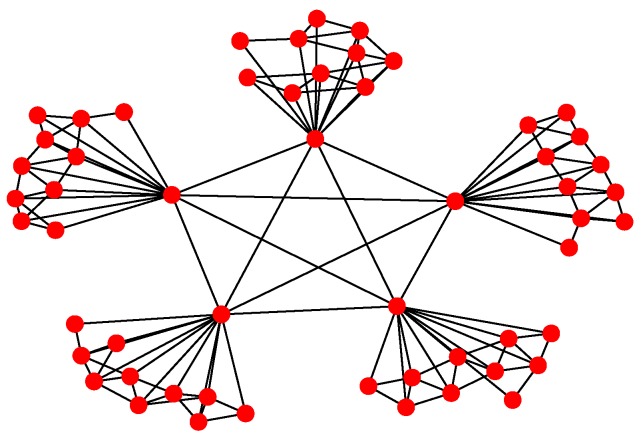
Communication layer of Structure 2.

**Figure 10:**
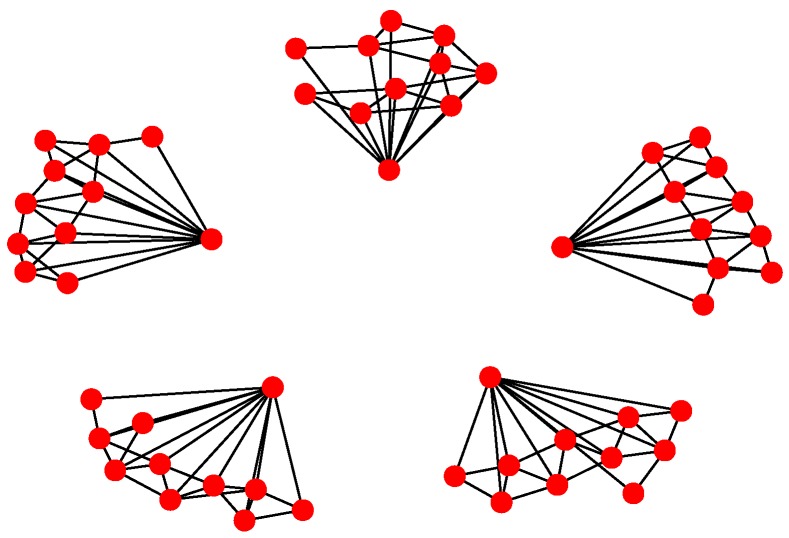
Structure layer of Structure 2.

**Figure 11:**
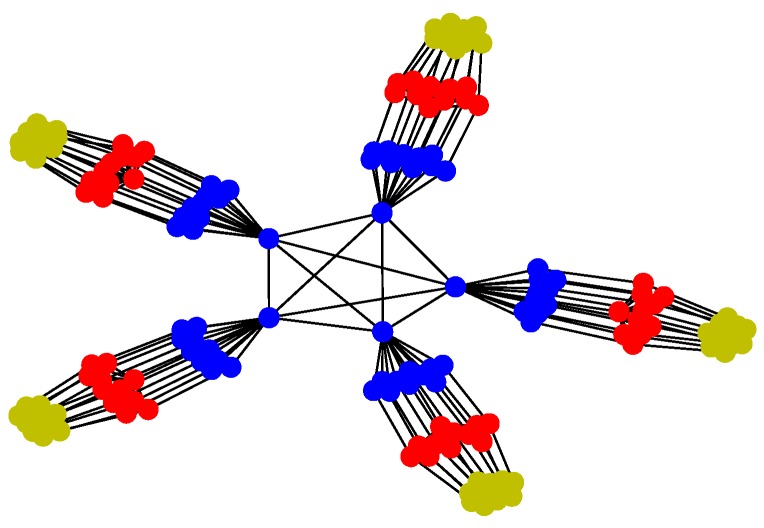
Network of Structure 2.

For Structure 2, suppose there are three leaders and each one has eleven followers in the UAV swarming system. Then, one can model the network with Algorithm 2. The communication layer is illustrated in Figure 9 and the structure layer has the same topology as the communication layer but no edges among the five leader nodes (see Figure 10). The mission layer is the same as in Structure 1; see Figure 7. The entire network of Structure 2 is shown in Figure 11.

**Figure 12:**
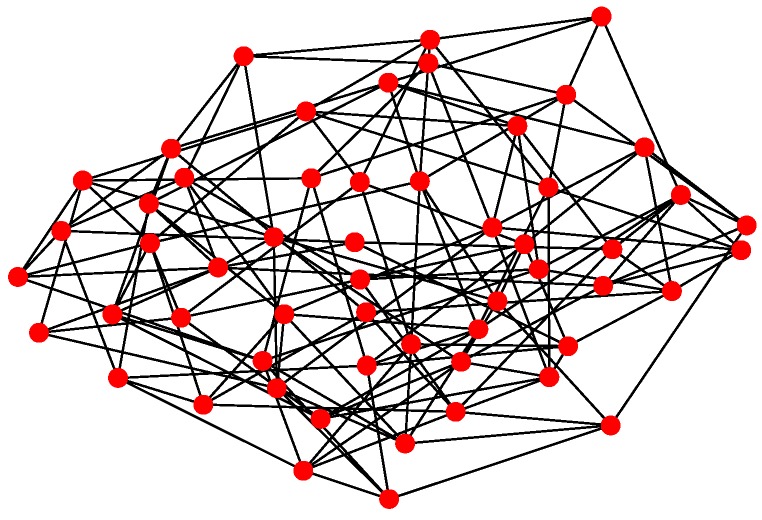
Communication and structure layers of Structure 3.

**Figure 13:**
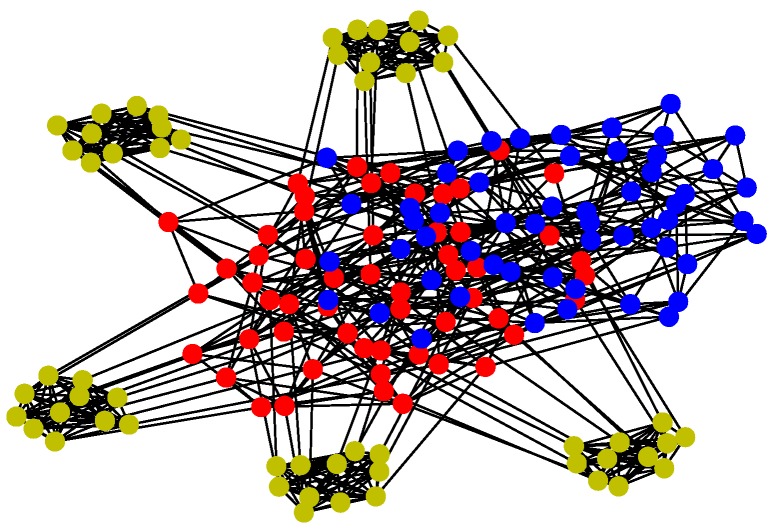
Network of Structure 3.

For Structure 3, let each vehicle connected with four to five adjacent ones consist of a swarming system with random topology. Therefore, the communication layer and the structure layer built by Algorithm 3 is shown in Figure 12. Then, based on the mission structure depicted in Figure 7, the network of Structure 3 can be seen in Figure 13.

### 4.2. Topology Analysis and Discussion

Analyzing the network topology is very important for two main reasons. Firstly, it can be used to estimate the characteristics of UAV swarming network and to obtain a deeper insight into the networks, specifically, note that most of the real-world networks have the features of a small-world or scale-free network. These two distinct topological properties have been studied for decades and have extensive mature theories and methods which can be used to guide the further research of the proposed network once some conditions are satisfied. Secondly, it provides a systematic way of understanding how the individual components fault affects the overall behavior or performance of the entire complex network and highlights the interaction and interdependency existed in the network. To this end, the static topology properties in the first three section will be analyzed in this section based on the network measurements presented in Section 3.3 and a reference basis for the dynamic topology analysis of the proposed network is also provided.

#### 4.2.1. Analysis of Degree and Degree Distribution of Nodes

**Figure 14:**
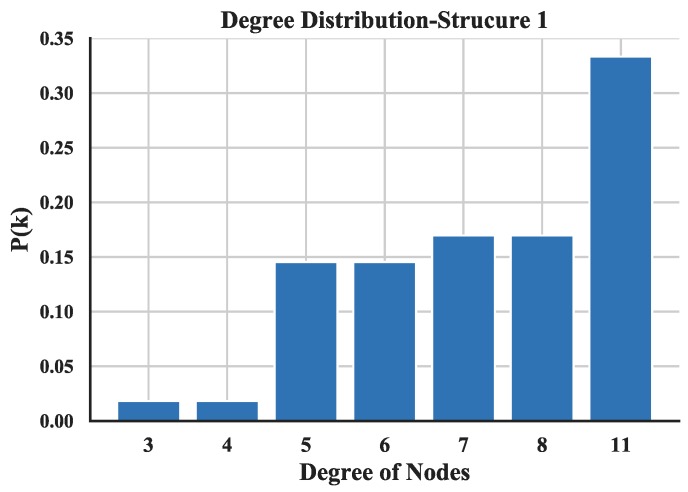
Distribution of degree of Structure 1.

**Figure 15:**
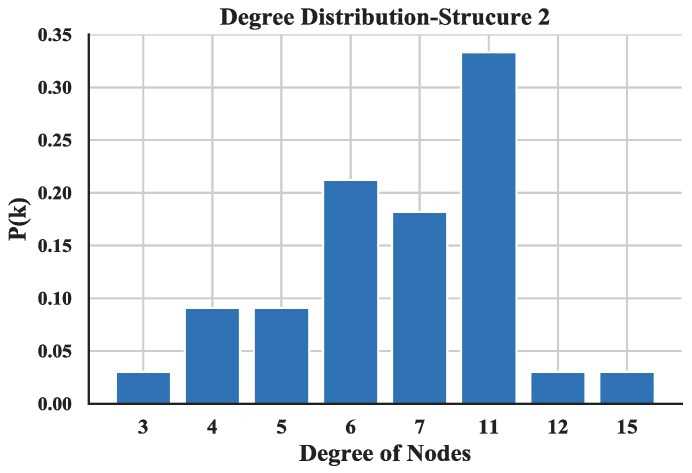
Distribution of degree of Structure 2.

**Figure 16:**
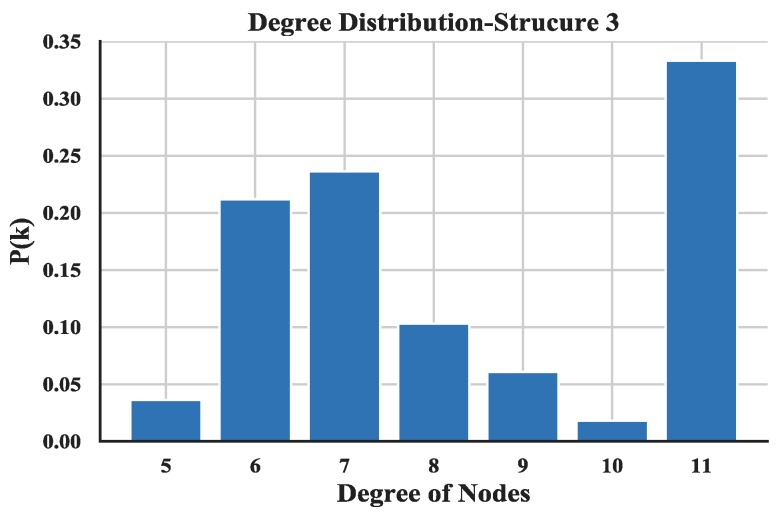
Distribution of degree of Structure 3.

The degree distribution reflects the connection over the entire network and provides a holistic view of the structure of UAV swarming network. The degree distributions of the three UAV swarming networks are shown in Figure 14, Figure 15 and Figure 16 and the average degrees are shown in Table 4. 

**Figure 17:**
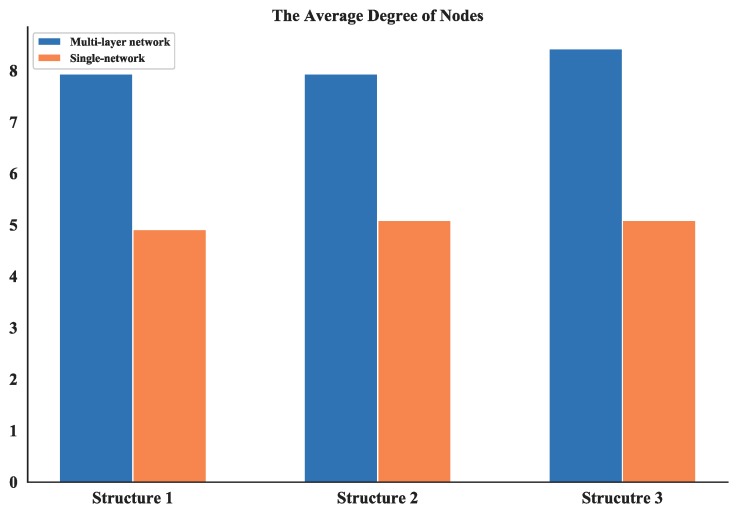
Comparison of average degree of nodes between multi-layer network and single network.

Note that most of the nodes connect more than 7 other nodes in the network and by looking at the average degree, the Structure 3 shows higher values; this is due probably to the random connection among the nodes. the average degree of nodes between multi-layer networks and single layer network are also compared. The results (see Figure 17) show that the proposed networks have higher degrees than the latter one.

Then, to identify the features of scale-free networks, the degree distribution of nodes is estimated to check whether it follows a power-law distribution, i.e.,

$$P\left(k\right)=ck^{-\lambda },\;$$

where *P*(*k*) denotes the proportion of nodes with a node degree of *k*, *c* is the coefficient, and *λ* is the power-law value.

**Figure 18:**
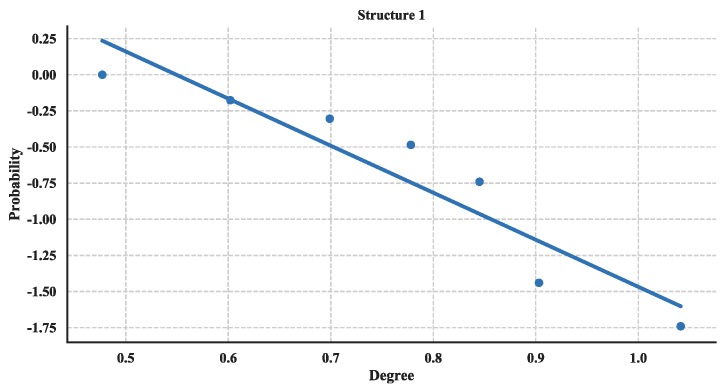
Cumulative Distribution of degree of Structure 1.

**Figure 19:**
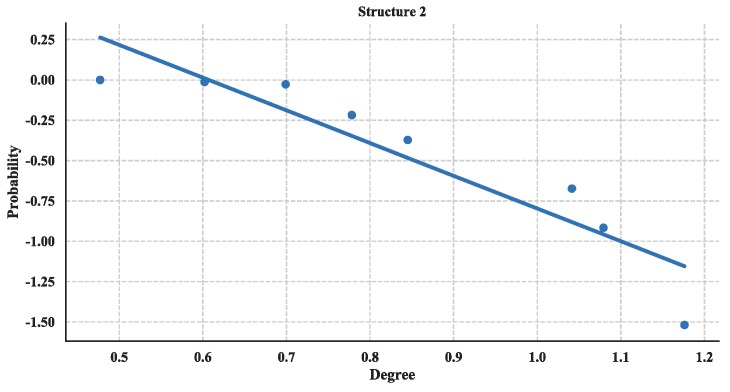
Cumulative Distribution of degree of Structure 2.

**Figure 20:**
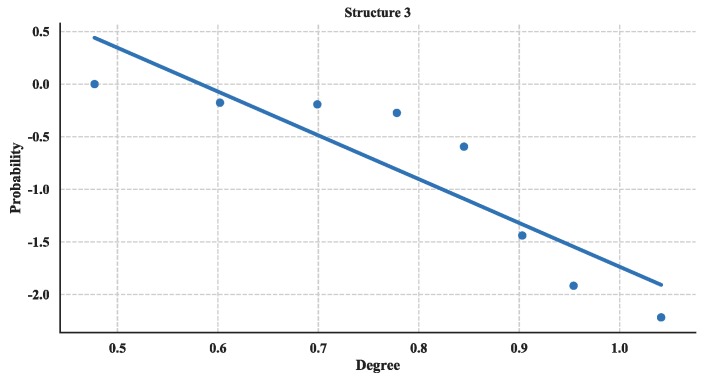
Cumulative Distribution of degree of Structure 3.

The distribution of degree is calculated with Equation (3) and then utilized a linear fitting method to fit these data and depict their logarithmic relationship. The results are illustrated in Figure 18, Figure 19 and Figure 20. Based on the results and from the perspective of a complex network, the node distributions of all three of the structures generally conform to a power-law decay function with *R*^2^ greater than 0.80. So, the UAV swarming networks have the topological properties of the scale-free network. One can know from the characteristic of the scale-free network that this network may be highly resistant to random attacks and be quite sensitive to targeted attacks [24].

#### 4.2.2. Analysis of Average Shortest Path Length

**Figure 21:**
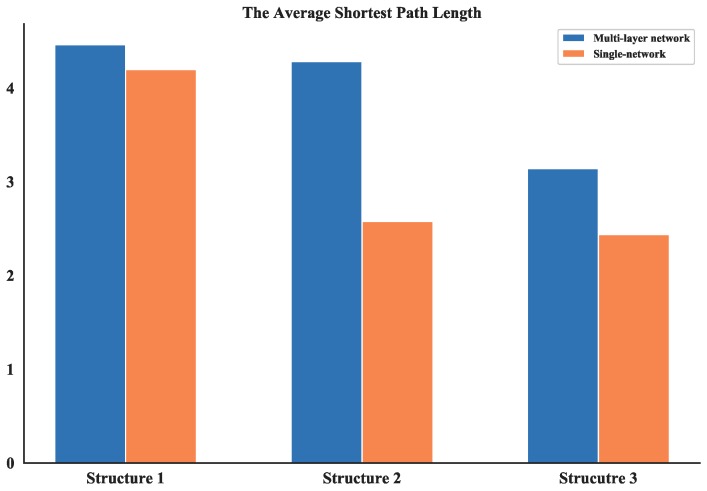
Comparison of average shortest path length between multi-layer network and single network.

The average shortest path length provides an indication of the convenience of traveling in a network as it quantifies the efficiency of the network in sending information between vertices [24]. the average shortest path length of all three UAV swarming networks are calculated and listed in Table 4 which shows the comparison results of these networks is: Structure 1 > Structure 2 > Structure 3 the value differences of average shortest path length between multi-layer networks and single layer network are also compared (see Figure 21). The average shortest path length of Structure 3 is also the smallest among all single layer networks. So, one can obtain that it is possible to have better communication in UAV swarms under autonomous control structure. 

#### 4.2.3. Analysis of Clustering Coefficient

**Figure 22:**
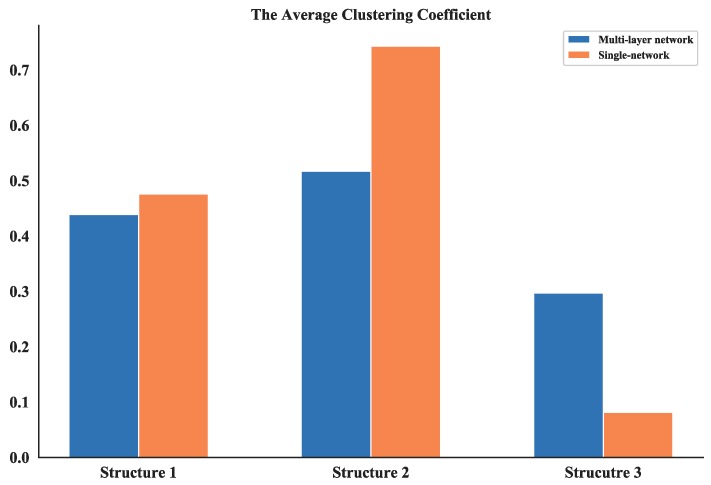
Comparison of average clustering coefficient between multi-layer network and single network.

The clustering coefficient of a node reflects the connectedness among its neighbors and the average clustering coefficient measures the global density of the interconnected nodes in a network. From the calculation results of average clustering coefficient of multi-layer networks and single networks (see Figure 22), it can be seen that the values of Structure 1 and Structure 2 of multi-layer networks are smaller than the single ones while the values of Structure 3 are the smallest one compare to all other networks. Since the single network can represent the communication layer of the multi-layer network, the result confirms that the communication in the first two structures is more concentrated. So, they could be easier to target during combat missions and less robust compared to Structure 3.

#### 4.2.4. Analysis of Small-World Characteristics

A small-world network refers to the size of the diameter of the network and also to the co-occurrence of a small diameter and a high clustering coefficient [48]. Thus, a small-world network can be judged by the following conditions:

$$\{\begin{array}{c} L\geq L_{random}\\ C\gg C_{random} \end{array}\mathrm{where}\{\begin{array}{c} L_{random}\approx \left(\ln N\right)/\left(\ln\left(<K>\right)\right)\\ C_{random}\approx <K>/N \end{array},\;$$

where *L* denotes the average length path, *C* is the clustering coefficient, and the subscript “random” denotes a random network.

To estimate if the networks built can meet the features of a small network, three random networks that have the same scale as the three structures are constructed. Then, the statistical features of these random networks are calculated and the results are listed in Table 5.

By comparing the clustering coefficients and average path lengths in Table 4 and Table 5, it can be seen that the clustering coefficients and average path lengths of the three types of UAV swarming networks are larger than those of the same-size random networks. Therefore, the three types of UAV cluster networks meet the small-world characteristics.

#### 4.2.5. Dynamic Topology Analysis

In many cases, the networks built by complex network theory can be taken as a basis for further estimating a network’s performance while under attack. This process is usually implemented by removing a node or multiple nodes in the network randomly or deliberately. These strategies have proved useful in the analysis of transportation networks [49,50], power grids [51], and critical infrastructure networks [52]. Herein, to prove the usefulness of the proposed multi-layer UAV swarming network for further analysis of robustness, a dynamic topology analysis is conducted by randomly removing a node in succession from the structure layer of the multi-layer networks and the single networks.

**Figure 23:**
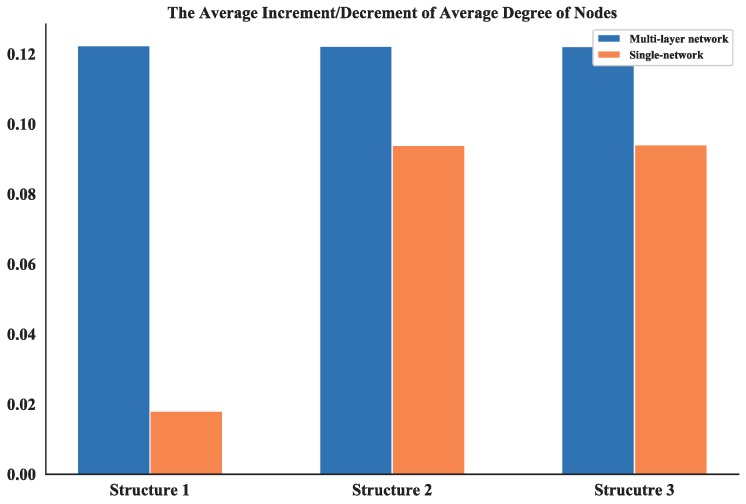
The average increment/decrement of average degree of nodes.

**Figure 24:**
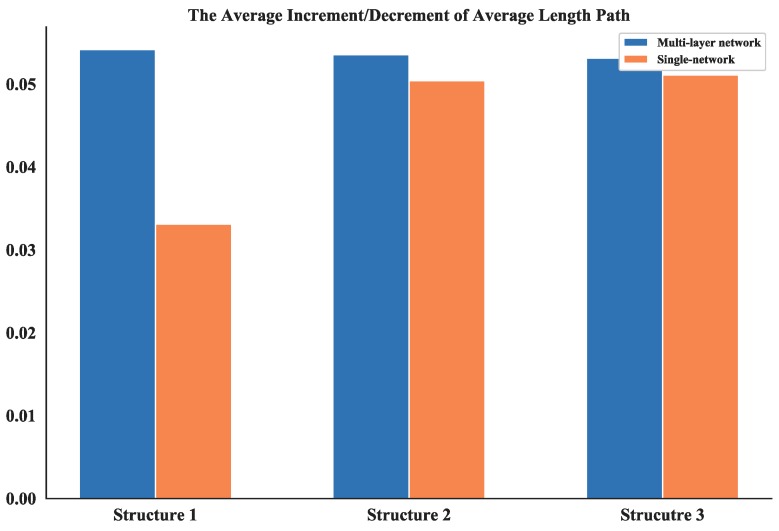
The average increment/decrement of average length path.

Then, based on the results of static topology analysis, one can see the values of average degree of nodes and average length path of the multi-layer network and single network show significant differences for all three structures. So, these two measurements in the dynamic topology analysis are chosen and their average increment or decrement are calculated. This process is simulated for 100 times and the absolute value of these average increments or decrements are shown in Figure 23 and Figure 24.

The average increment/decrement of these measurements demonstrate that the multi-layer network is more sensitive to the nodes changing than the single network. So, the proposed network could be more useful in the analysis of robustness and make it easier to estimate the measurements.

**Figure 25:**
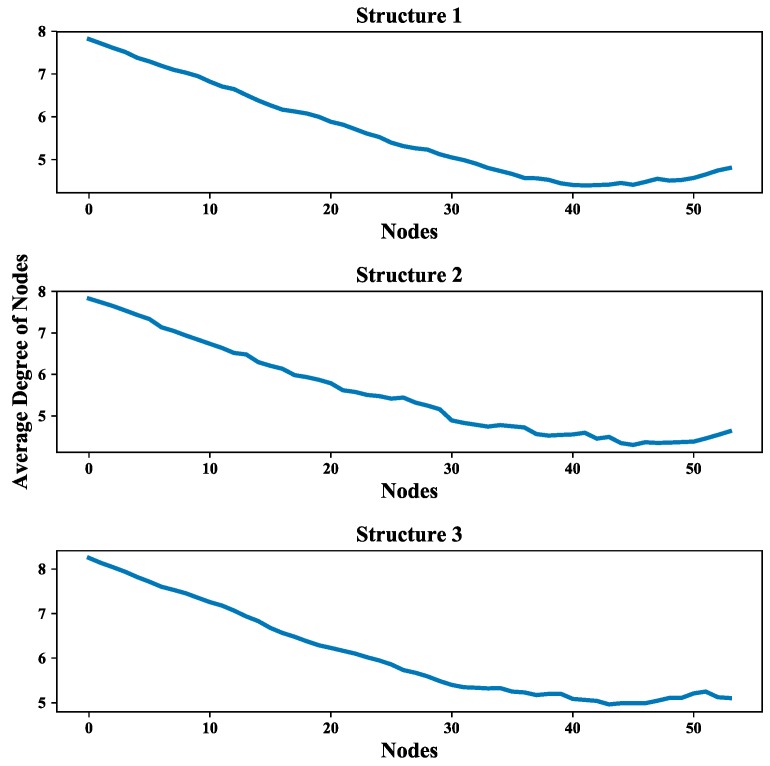
Effect of node removing on values of average degree of nodes.

**Figure 26:**
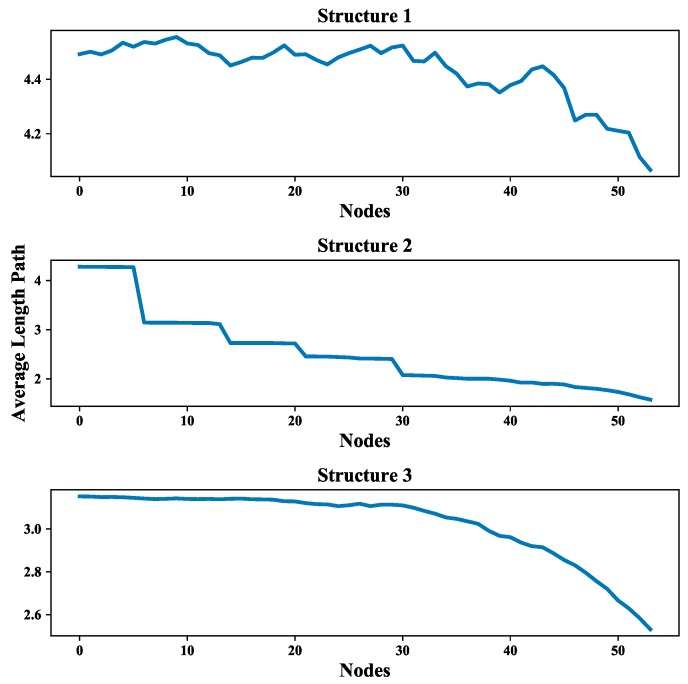
Effect of node removing on values of average length path.

Moreover, the multi-layer UAV swarming networks also provide a framework to simulate the mission scenario like the communication in the system is blocked and further affect the mission. This scenario is presented by randomly removing nodes from the communication layer of the proposed network. The effect of node removing on the average degree of nodes and average length path of these three structures are shown in Figure 25 and Figure 26. The average increment/decrement of average degree of nodes and average length path are shown in Table 6.

It can be seen from Figure 25 and Figure 26, the values of these two network measurements depredate with the removing of nodes and the degradation process is quite steady and clear. One can also see from Table 6 that the average increment/decrement of them are suitable for further analyzing the communication network as well as the UAV swarming network. These results show that this framework is valid in the study of inter-dependent relations among communication layer, structure layer and mission layer and also can be improved by considering the cascade failures in communication networks reference to the relevant research literature [53,54]. 

### 4.3. Robustness Evaluation

Based on the features of a network and the topology analysis results above, one can present the robustness metrics of network. These metrics will be much more suitable for analyzing the system. Efforts have been carried out to study how to define new measurements. Manzano, for example, introduced a measure called “endurance” and quantified the level of robustness supported by a specific topology under different types of multiple failure scenarios [55]. Van Mieghem proposed an *R*-value, ranging from 0 to 1, to assess the robustness of any network [56]. 

Here, a robustness metric can be present to estimate the network robustness by using the existing studies for reference (see [56] for details), it can be written as:

(7) $$R=\sum_{k=1}^{m}s_{k}t_{k},\;$$

where *s* and *t* are the weight and network measurement, respectively. The weight can be determined by considering the importance of the corresponding measurements for the network. The *R* will then be normalized to the interval [0,1], and *R* = 1 reflects complete robustness.

Then, known from the topology analysis results, the average degree of nodes and average length path can reflect the robustness of network both when nodes changing on structure layer and communication layer of the network. To simplify this example, the weight is set to be 1 for both measurements, so (7) can be written as;

(8) $$R=t_{\deg ree}+t_{path},\;$$

where *t_degree_* and *t_path_* are the average degree of nodes and average length path, respectively.

**Figure 27:**
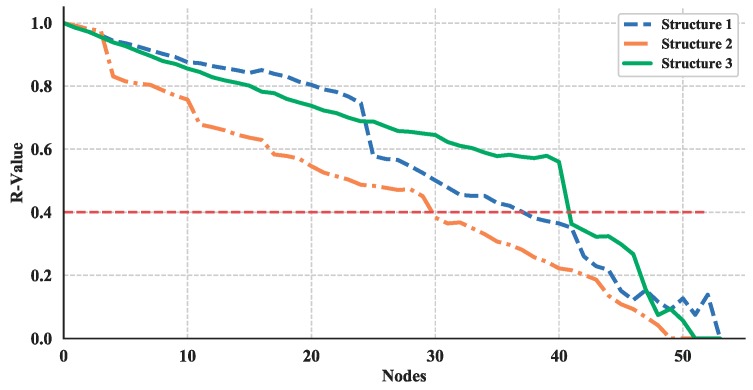
Robustness illustration, where nodes removed from structure layer of network.

**Figure 28:**
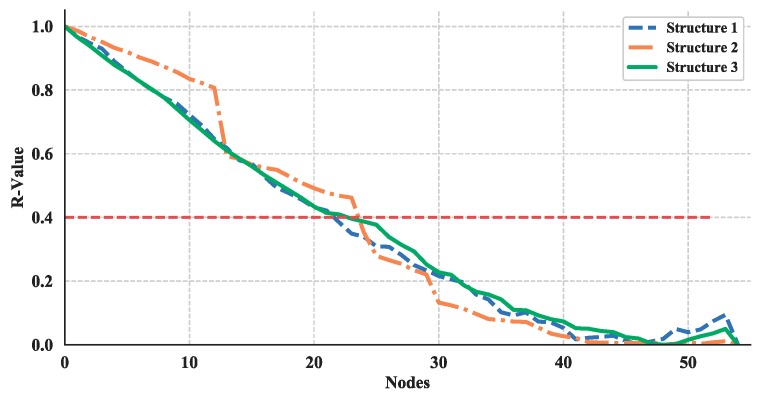
Robustness illustration, where nodes removed from communication layer of network.

Thus, the effect of node removing on the robustness of these three structures are shown in Figure 27 and Figure 28. The red-dotted line denotes the failure threshold of the UAV swarming network. If the value is set to 0.4, then one can see from Figure 27 and Figure 28 the corresponding nodes number are a minimum of 30 for nodes removed from structure layer of network and less than 30 for communication layer. Based on the results, one can say that the communication layer is more fragile than structure layer and need more protection.

## 5. Conclusions and Future Work

Considering the new requirements for the robustness and reliability of a UAV swarming system, a system modeling framework is proposed to gain a full understanding of the system and is taken as a basis to enable further analysis of a UAV swarming system. To achieve this, a generalized description of a UAV swarming system based on a sufficient survey is provided. The scope of work from the point of view of control algorithms is also identified considering the dynamics and research novelty of the development of a UAV swarming control algorithm. The developed framework offers three algorithms of three control structures, each consisting of three inter-dependent network layers: communication, structure, and mission layers. Some network measurements obtained by adjusting the fundamental ones into a UAV swarming system are also introduced. The case study illustrated the visualization models and estimated the statistical characteristics of the proposed networks based on these measurements. The results confirmed that the networks built in the case study have the characteristics of a small-world and scale-free network. The dynamic topology analysis and a simple demonstration of robustness evaluation also confirms that this framework may allow us to further analyze the robustness or reliability of a UAV swarming system in a high-confrontation battlefield environment and the effect of cascading failure in an ad hoc network on the system.

In the future, we plan to use the proposed framework to develop networks whose robustness will be investigated by estimating their statistical features while under random and targeted attacks. Two characteristics of a UAV swarming system, cascading failures in the communication network and dynamic reconfiguration of the entire system, will be considered. An optimization method of the confrontation strategy considering robustness, cost, and resources will also be explored.

## Figures and Tables

**Table 1:** Modeling algorithm for Structure 1.

**Algorithm 1: Modeling Algorithm for the control structure of Behavior-based methods**
**1: Initialization: *V_a_*[1…*n*]*, V_b_*[1…*n*]*, V_c_*[1…*n*], *n*, *m*, *n*[1…*m*],** **2: for *i*←1 to *n* do** **3: addedge&weight [(*V_a_*[i], *V_b_*[i], weight), (*V_b_*[i], *V_c_*[i], weight)]** **4: for *x*←1 to *p* do** **5: for *j*←1 to *x* do** **6: addedge&weight[(*V_a_*[i], *V_a_*[i+*x*], weight), (*V_b_*[i], *V_b_*[i+*x*], weight)]** **7: addedge&weight [(*V_a_*[i], *V_a_*[i+*x*+1], weight), (*V_b_*[i], *V_b_*[i+*x*+1], weight)]** **8: addedge&weight [(*V_a_*[i+*x*], *V_a_*[i+*x+*1], weight), (*V_b_*[i+*x*], *V_b_*[i+*x+*1], weight)]** **9: end for** **10: end for** **11: end for** **12: for *i*←1 to *m* do** **13: for *j*←1 to *n*[*i*] do** **14: for *x*←*j* to *n*[*i*] do** **15: addedge&weight (*V_c_*[*j*], *V_c_*[*j*+*1*], weight)** **16: end for** **17: end for** **18: end for** **19: return G(*G_a_*, *G_b_*, *G_c_*)**

**Table 2:** Modeling algorithm for Structure 2.

**Algorithm 2: Modeling Algorithm for the Control Structure of Leader-Follower Strategy**
**1: Initialization: *V_a_*[1…*n*], *V_b_*[1…*n*], *V_c_*[1…*n*], *n*, *m*, *n*[1…*m*]** **2: for *i*←1 to *n* do** **3: addedge&weight [(*V_a_*[*i*], *V_b_*[*i*], weight), (*V_b_*[*i*], *V_c_*[*i*], weight)]** **4: end for** **5: for *i*←1 to *m* − 1 do** **6: addedge&weight (*V_a_*[*i*], *V_a_*[*i*+1], weight)** **7: end for** **8: for *i*←1 to *m* do** **9: for *j*←1 to *n*[*i*]-1 do** **10: addedge&weight [(*V_a_*[*i*], *V_a_*[*j*], weight), (*V_b_*[*i*], *V_b_*[*j*], weight)]** **11: end for** **12: for *x*←*j* to *n*[*i*] do** **13: addedge&weight (*V_c_*[*j*], *V_c_*[*j*+1], weight)** **14: end for** **15: for *j*←1 to *n*[*i*]-3 do** **16: addedge&weight [(*V_a_*[*j*], *V_a_*[*j*+1], weight), (*V_b_*[*j*], *V_b_*[*j*+1], weight)]** **17: addedge&weight [(*V_a_*[*j*], *V_a_*[*j*+2], weight), (*V_b_*[*j*], *V_b_*[*j*+2], weight)]** **18: end for** **19: end for** **20: return *G*(*G_a_*, *G_b_*, *G_c_*)**

**Table 3:** Modeling algorithm for Structure 3.

**Algorithm 3: Modeling Algorithm for the Autonomous Control Structure**
**1: Initialization: *V_a_*[1…*n*]*, V_b_*[1…*n*]*, V_c_*[1…*n*], *n*, *m*, *n*[1…*m*], *k*** **2: for *i←*1 to *n* do** **3: addedge&weight [(*V_a_*[*i*], *V_b_*[*i*], weight), (*V_b_*[*i*], *V_c_*[*i*], weight)]** **4: end for** **5: for *i←*1 to *k* do** **6: for *j←*1 to *m* do** **7: randomly choose nodes *V_a_*, *V_b_*** **8: if *V_a_*, *V_b_*≠ *V_a_*[*j*], *V_b_*[*j*] then** **9: addedge&weight [(*V_a_*, *V_a_*[*j*], weight),(*V_b_*, *V_b_*[*j*], weight)]** **10: end if** **11: end for** **12: end for** **13: for *i←*1 to *m* do** **14: for *j←*1 to *n*[*i*]-1 do** **15: for *x←j* to *n*[*i*] do** **16: addedge&weight (*V_c_*[*j*], *V_c_*[*j*+1], weight)** **17: end for** **18: end for** **19: end for** **20: return G(*G_a_*, *G_b_*, *G_c_*)**

**Table 4:** Statistical features of three UAV swarming networks.

Features	Structure 1	Structure 2	Structure 3
Number of nodes	165	165	165
Number of edges	655	655	695
Average degree	7.94	7.94	8.42
Cluster coefficient	0.44	0.52	0.30
Average length path	4.46	4.28	3.14

**Table 5:** Statistical features of three random networks.

Features	Random Network 1	Random Network 2	Random Network 3
Number of nodes	165	165	165
Number of edges	655	655	695
Cluster coefficient	0.03	0.03	0.04
Average length path	2.68	2.68	2.60

**Table 6:** The average increment/decrement of features.

Features	Structure 1	Structure 2	Structure 3
Average degree of nodes	−0.06022	−0.06027	−0.06023
Average length path	−0.04863	−0.04866	−0.04886
